# Dynamic balance in patients with degenerative lumbar spinal stenosis; a cross-sectional study

**DOI:** 10.1186/s12891-018-2111-x

**Published:** 2018-06-15

**Authors:** Elisabeth Thornes, Hilde Stendal Robinson, Nina Køpke Vøllestad

**Affiliations:** 1 Department of Physical Therapy, Martina Hansens Hospital, PO.Box 823, 1306 Sandvika, Norway; 2 Department of Health Sciences, University of Oslo, Oslo, Norway

**Keywords:** Disability, Assessment, Balance, Rehabilitation

## Abstract

**Background:**

Degenerative lumbar spinal stenosis (LSS) is a prevalent condition in adults over the age of 55 years. The condition is associated with activity limitations that are related to increased pain when engaging in weight-bearing activities, such as walking and standing, and release of pain while sitting down or bending forward. The limitation on ambulation is also associated with impaired balance although the types of balance problems are sparsely described in this patient group. The purpose of this study was to assess dynamic balance in persons with LSS by the Mini-BESTest and explore the associations with self-reported balance and functional disability.

**Methods:**

Sixty two participants were included in this cross-sectional study. The main outcome measure was the Mini-BESTest, providing a total score and sub-scores for 4 balance control systems (Anticipatory Adjustment, Reactive Response, Sensory Orientation, Stability of Gait). The Swiss Spinal Stenosis Questionnaire provided sub-scores for self-reported balance problems and walking function (FUNC).

**Results:**

The participants showed large inter-individual variation in all measures of balance. The Mini-BESTest score ranged from very good to poor and the mean value was 22.8 (SD 3.5). Nineteen participants (31%) reported having frequent balance problems. Logistic regression analyses showed that both the total Mini-BESTest score (OR (95% CI) 1.6 (1.2, 2.0)(*P* = .001) and 3 of the 4 balance control systems (Anticipatory Adjustment, Sensory Orientation, Stability in Gait) were significantly associated with self-reported balance problems (.001 ≤ *P* ≤ .01). The strongest association was seen between Sensory Orientation and balance problems, implying that it is 4.4 times more likely that persons would have no or occasional balance problems with each unit of increase in Sensory Orientation. The total score for the Mini-BESTest was significantly associated with FUNC (*P* = .042).

**Conclusions:**

The dynamic balance of persons with LSS showed a large heterogeneity with a large fraction of the participants displaying no balance impairments. The test results were associated with the participants’ self-reported balance problems and walking function. The Mini-BESTest thus appears to provide additional information to self-reported disability, and by identifying different kind of balance control impairments, the Mini-BESTest could be useful for physiotherapists working with person-centered rehabilitation in persons with LSS.

**Electronic supplementary material:**

The online version of this article (10.1186/s12891-018-2111-x) contains supplementary material, which is available to authorized users.

## Background

Degenerative lumbar spinal stenosis (LSS) is a common condition among older adults [1]. The main functional problem for persons diagnosed with LSS is walking limitations due to increased pain and/or paresthesia in their feet as a result of prolonged walking [2, 3]. These individuals also commonly report problems with balance [4–8]. Based on single performance tests of balance, previous studies have identified balance limitation in 40–65% of persons with LSS [9–11]. Hence, a substantial fraction appears to have no problems compared to population of same age. Observations of wide-based gait, which is one of the characteristics of persons with LSS [10], indicate that their dynamic balance is also limited [12]. In a recent study, we showed that a relatively high proportion of persons with LSS were unable to complete the tandem walk (39%) and the one-leg stance (weight-transfer from 2 legs to a single leg) (58%), while relatively few were unable to complete the Romberg maneuver (7%) [6]. Moreover, the results indicate a large variation in balance problems among LSS patients. Dynamic balance limitation is also documented in the study by Kim et al., who conclude that persons with LSS have a higher risk of falling than persons with osteoarthritis in the knees [13]. Furthermore, Passmore et al. found that persons with LSS spent more time planning and performing challenging lower limb movements than healthy adults [14].

Although balance problems are common among persons with LSS, the disability has received little attention in guidelines for assessment and treatment [15–18]. Conservative management, which is recommended as the initial treatment, includes widely varying approaches, such as strength training, coordination and endurance exercises, stretching, mobilization, and patient education and counseling. A systematic assessment is essential for the management of such persons’ balance problems in clinical practice. A key factor in person-centered decisions is to identify the patient’s individual limitations and impairments. Previous studies of balance in persons with LSS have either used single physical performance measures [6, 9] or advanced technical equipment [14]. The introduction of “The Balance System Evaluation Test”, developed by Horac and co-workers, is frequently used in assessing balance disability in older adults and persons with Parkinson’ disease or stroke [19–23]. This comprehensive test assesses the underlying mechanisms in the balance control systems, and the test results can be useful in selecting interventions in the rehabilitation process of persons who have different limitations on postural responses, including patients with LSS. The original test was rather time-consuming and consisted of 36 items, grouped into 6 control systems. It was developed to assess balance in persons with neurological diseases. A shortened version, the Mini-BESTest, with 14 items/tests, was presented in 2010 [24]. These 14 tests primarily assess dynamic balance reflecting balance challenges during everyday activities and provide a total score as well as 4 sub-scores for the different balance control systems: 1) the anticipatory activation system (the ability to pre-activate the musculoskeletal system when planning a movement), 2) reactive responses (the ability to counteract external disturbances), 3) sensory orientation (proprioceptive and visual adjustment in postural control), and 4) stability in gait (the ability to perform dual tasks, e.g., to cross an obstacle, turn the head, or count while walking). Since the Mini-BESTest captures limitations in the different underlying balance control systems, we hypothesized that the test could be used to provide new understandings of balance in persons with symptomatic LSS, and that the responses could be useful in planning relevant treatment for the persons with LSS.

This study had three objectives. The first was to assess dynamic balance in a group of persons with LSS using the Mini-BESTest. The second objective was to examine how the four balance control systems relate to the self-reported frequency of balance problems. The third objective was to examine the associations between dynamic balance and self-reported measures of function and disability.

## Methods

### Participants

During 2013, 62 participants with MRI-verified LSS were recruited from an outpatient clinic in a hospital in specialized health care in Norway. To be eligible for inclusion to the study the person had to be older than 55 years, understand oral and written Norwegian, and have symptomatic and radiologically verified degenerative central LSS. Participants were excluded from participation if they had co-morbidities that could affect their physical performance, such as neurological or rheumatologic diseases, painful osteoarthritis in the lower limbs, vestibular-neuritis, or peripheral vascular diseases. After being identified from the hospital’s waiting lists, the participants received an information letter with an invitation to participate in the study. After giving oral approval by telephone, the participants received and completed a written consent form. The local hospital board and the Regional Ethics Committee approved the study (REK: 2010/2907a), as the human rights of the participants were protected.

**Fig. 1:**
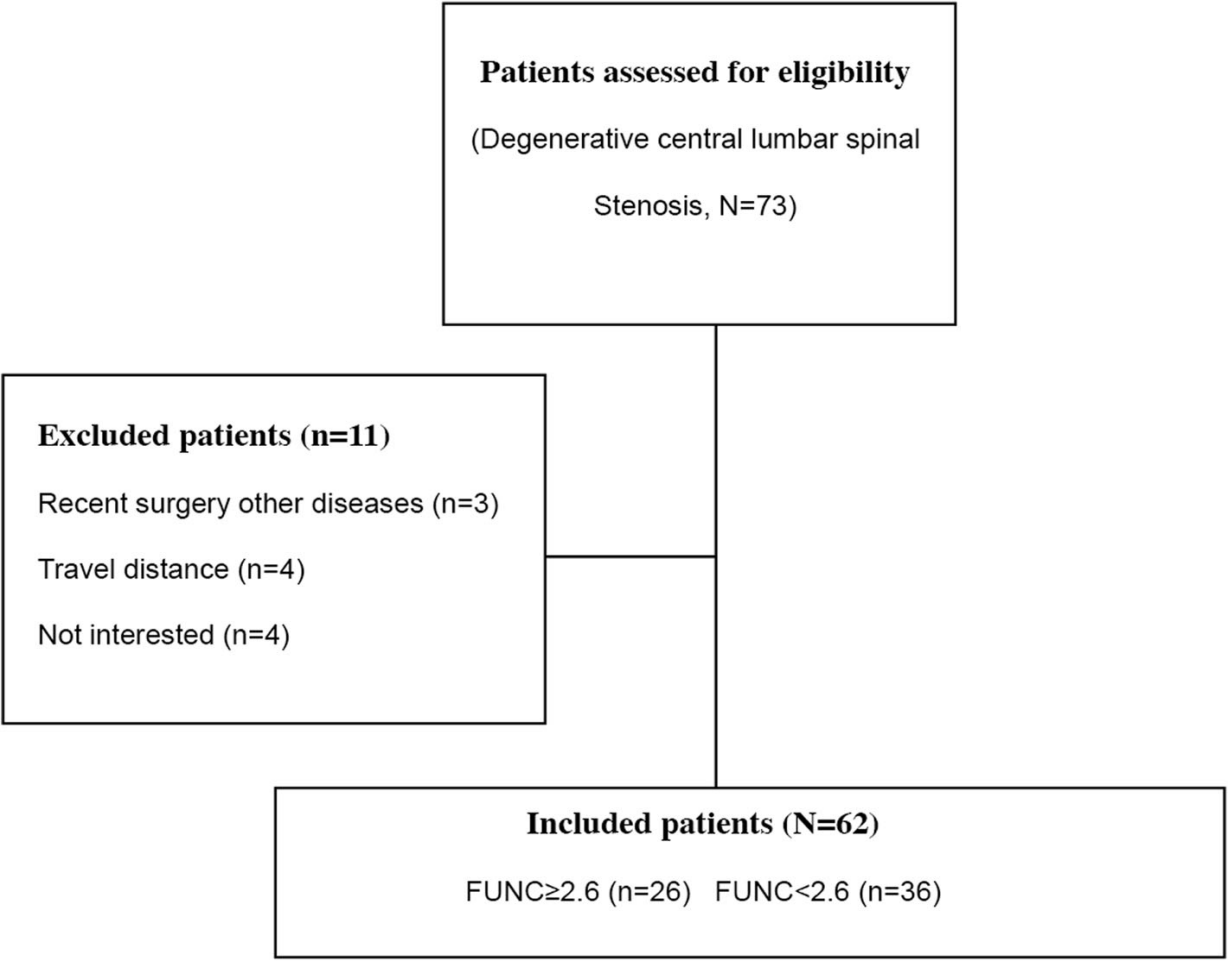
Flow-chart and patient distribution

To ensure broad representation of the patient population, we consecutively included participants a) referred to surgery and poor physical function, b) referred to surgery and moderate physical function, and c) not referred to surgery. Physical function was assessed using the Physical Functioning (FUNC) scale from the Swiss Spinal Stenosis Questionnaire (see below). Poor physical function was defined as FUNC≥2.6 and moderate was defined as FUNC< 2.6 [25]. We aimed for a sample size of at least 60 participants [26] (Fig. 1).

### Procedure

The participants completed a comprehensive questionnaire before the physical assessment, including questions about personal characteristics such as gender, age, weight, height, smoking habits, and duration of actual low back pain and lower limb pain. All participants were tested by one of three research assistants at the hospital, blinded for disability level and appraisal for surgery, who were trained in using the test protocol. Both participants and assistants were instructed not to discuss the following treatment. Consensus was reached among the assessors on how to conduct the tests before inclusion started. Each test session took approximately 20 min.

### Outcome measures

#### The mini-BESTest

The test consists of 14 tasks covering dynamic control in maintaining posture/balance, scored on a 3-level ordinal scale (Table 1) [24, 27, 28]. The total score range is 0–28, with the highest score understood as normal/good postural control. A cut-off of 21 is reported to differentiate between people with and without impaired postural responses [29].

**Table 1:** The 14 items forming the Mini-BESTest and the items belonging to the 4 subsystems in balance control

I. Anticipatory Postural Adjustments	II. Reactive responses	III. Sensory Orientation	IV. Stability in gait
1. Sit-to-stand 2. Rise-to-toes 3. Stance on one leg (20s)	4. Compensatory stepping correction forward 5. Compensatory stepping correction backward 6. Compensatory stepping correction lateral	7. Stance on firm surface, eyes open 8. Stance on foam, eyes closed 9. Stance on incline, eyes closed	10. Change in gait speed 11. Walk with head turns, horizontal 12. Walk with pivot turns 13. Step over obstacles 14. Timed Up and Go with dual task

Scores range from 0 to 2 at each item, where 2 is normal balance, 1 is moderate loss of normal postural responses and 0 is loss of normal postural responses. Where two sided (3, 6, 14), worse response is used. Total score range: 0–28

The reliability of the Mini-BESTest, both inter-rater and test-retest reliability, is reported to be excellent, with ICCs of 0.98 (0.97–0.99) and 0.96 (0.94–0.99), respectively [30]. The content validity is also reported to be high [24, 31]. The minimal clinically important difference (MCID) is 4 points [30]. The sub-scores for the items in each of the 4 control systems range from 0 to 6 (Anticipatory Adjustment, Reactive Response, and Sensory Orientation) and from 0 to 10 (Stability in Gait).

#### Self-reported measures

The comprehensive questionnaire also consisted of a cluster of validated self-reported measures. The disease-specific signs were assessed using the Symptom Severity (SYMP) and FUNC scales, 2 of 3 subscales in the Swiss Spinal Stenosis Questionnaire (SSQ) [32]. The seven questions in SYMP addresses overall pain, pain frequency, pain in the back, pain in the leg, numbness, weakness and balance disturbance and is scored as an un-weighted mean, range 1–5 (1 = no symptoms, 2 = mild symptoms, 3 = moderate symptoms, 4 = severe symptoms, 5 = very severe symptoms). FUNC has 5 questions asking about walking distance and ability to walk for pleasure, for shopping, and getting around in the house/apartment, and from bathroom to bedroom. The score range is 1–4 (1 = Yes, comfortably; 2 = yes, sometimes with pain; 3 = yes, always with pain; 4 = no, cannot perform).

Pain-related disability was measured using the Oswestry Disability Index (ODI), with a score range of 0–100 (100 = worst) [33]. The ODI evaluates the impact of back pain on daily living activities [34].

Pain intensity was recorded using 2 numeric rating scales (NRS) for lumbar and lower limb pain, respectively, (0–10, where 10 = worst imaginable pain). Health-related quality of life was assessed using the European Quality of Life Questionnaire,(EQ5D) providing a utility measure (0–1, where 0 = worst) [35]. Hopkins Symptom Checklist (HSCL-25) was used to examine distress (1–4, where 4 = worst) [36].

### Data analyses

The participants’ characteristics and results from the Mini-BESTest are described by frequencies, percentages, means with standard deviations (SD) and minimum-maximum (Min-Max) where appropriate. The 7th item of the SYMP scale is a question about balance: “In the last, how would you describe problems with your balance? Yes, often I feel my balance is off, or that I am not sure-footed/ Yes, sometimes I feel my balance is off, or that I am not sure-footed/ No, I’ve had no problems with balance?”^32^ Based on the response on this question, the participants were divided into 3 groups. Group 0: Participants without balance problems; Group 1: Participants with balance problems sometimes; and Group 2: Participants with balance problems often.

Univariate analyses of variance were carried out to assess differences between the three balance groups (Group 0–2) with Games-Howell post hoc tests The associations between the self-reported and the performance-based variables were assessed by bivariate correlations (Spearman’s Rho). The strength in the correlation are defined as weak (.10–.29), medium (.30–49) and large (.50–1.0) [37].

Binary logistic regression analyses were used to explore the associations between balance problems and the Mini-BESTest, and each of the 4 control systems separately. For these analyses, a dichotomous variable (Balance_2) was constructed, merging Group 0 and 1 (coded 1), leaving those with frequent balance problems in a separate group (coded 0). Associations between FUNC and ODI as dependent variables and the Mini-BESTest results were examined using linear regression models. Age, gender, and body mass index (BMI) were entered as covariates in the adjusted models, since these variables have previously been shown to be associated with ambulatory ability [38], physical function [39], and the condition of clinical LSS [40].

SPSS (IBM) version 23 was used in all analyses and a value of *P* < .05 was used as the significance level.

## Results

### Participants

In all, 62 participants with LSS, 47% of them female, were included in the study. The mean (min-max) age was 71.2 (56–83) years and mean BMI (kg/m^2^) was 28.5 (19–42). The participants displayed large variation in all self-reported measures of symptoms and disability (Table 2), with no gender differences (*P* > .08); 19 of the participants had frequent balance problems (Group 2), whereas 29 had balance problems sometimes (Group 1), and 14 participants reported having no balance problems (Group 0).

**Table 2:** Patient characteristics presented for all patients and the three balance groups (Group 2 = often balance problems, Group 1 = sometimes balance problems, Group 0 = no balance problems)

Variables	All patients		Group 2		Group 1		Group 0		*p*-value^a^
	*N* = 62		*N* = 19		*N* = 29		*N* = 14		
	n	%	n	%	n	%	n	%	
Gender, female/male	29/33	(47/53)	8/11 (42/58)	(42/58)	15/14	(52/48)	6/8	(43/57)	.764
	Mean (SD)	Min-Max	Mean (SD)		Mean (SD)		Mean (SD)		p-value^b^
Age, y	71.2 (7.1)	56-83	71.1 (7.5)		72.4 (7.2)		70.6 (6.7)		.909
BMI , kg/m^2^	28.5 (4.9)	19-42	30.2 (5.6)		28.7 (4.4)		25.9 (4.6)		.036
SYMP, 1-5	3.2 (0.7)	1.3-4.6	3.6 (0.4)		3.3 (0.6)		2.5 (0.6)		<.001
SYMP_6, 1-5	3.2 (0.7)	1.3-4.8	3.4(0.5)		3.2 (0.7)		2.8 (0.7)		.034
FUNC,1-4	2.3 (0.6)	1.0-3.4	2.4 (0.5)		2.4 (0.6)		1.9 (0.7)		.028
ODI, 0-100	31.0 (13.4)	0-60	30.1 (9.1)		32.1 (14.6)		23.1 (14.8)		.034
Lumbar pain, 0-10	5.2 (2.8)	0-10	5.2 (2.8)		6.2 (2.7)		3.3 (2.3)		.004
Lower limb pain, 0-10	5.9 (2.6)	0-10	6.1 (2.3)		6.4 (2.4)		4.6 (3.1)		.119
EQ5D, 0-1	0.6 (0.23)	0.02-1	0.6 (02)		0.5 (0.2)		0.7 (0.2)		.212
HSCL 25,1-4	1.4 (0.3)	1-2.2	1.5 (0.3)		1.4 (0.3)		1.3 (0.2)		.124

^a^Pearson chi-square two-sided, ^b^univariate analyses of variance (GLM) *SYMP* Spinal stenosis questionnaire , Symptom Severity , *SYMP_6* SYMP without the balance question. *FUNC* Spinal Stenosis Questionnaire; physical function, *ODI* Oswestry Disability Index, *NRS* Numeric rating scale, *EQ5D* European Quality of Life Utility Score, *HSCL25* Hopkins Symptom Check List 25

Statistical differences between the groups were found for BMI, FUNC, SYMP, ODI, and lumbar pain (0.001 < *P* ≤ 0.036) (Table 2). For all these variables, Group 2 displayed the lowest mean values, whereas no systematic differences were observed between Groups 0 and 1. For the total score of the Mini-BESTest, statistically significant differences were found between the participants in Gr2 and the two other groups; for Gr1 *P* = .032 and for Gr0 *P* = .006. There were no statistical differences between the groups in relation to age, gender, lower limb pain, EQ5D, or HSCL25 (*P* > 0.1). The participants showed low mental distress with mean HSCL25 scores of 1.5 or lower in all 3 groups.

### Dynamic balance assessed by the mini-BESTest

**Fig. 2:**
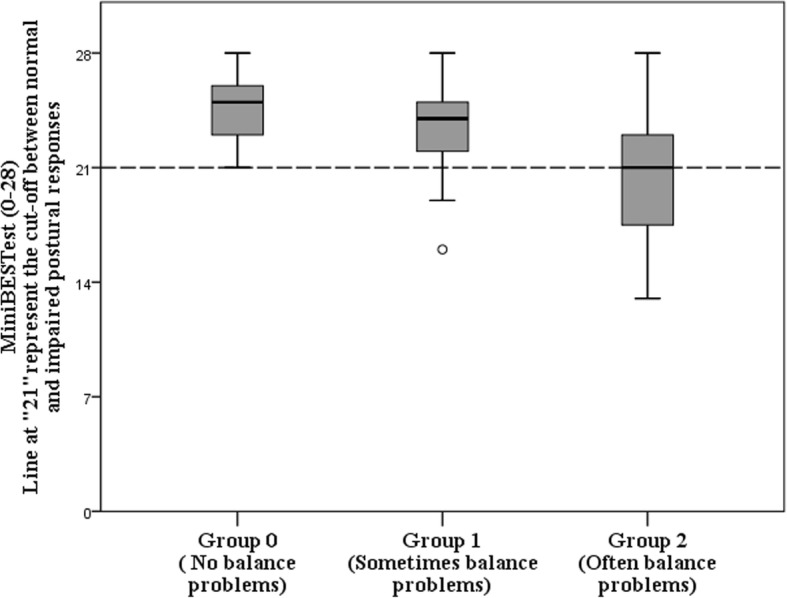
Boxplot of Mini-BESTest scores for the 3 Balance groups

All participants completed the tests safely without falling, and no participant asked for the testing to be stopped or needed walking aid. The mean (SD) score for all participants in the Mini-BESTest was 22.8 (3.5). In all 3 balance groups, at least 50% scored above 21 and were thus within the normal range (Fig. 2).

However, statistically significant group differences were found for the total score on the Mini-BESTest (*P* = .002). The female participants scored significantly more poorly than male participants (mean difference − 1.8, and 95%CI -3.5,-0.1), *P* = .038.

For 3 of the control systems: Anticipatory Adjustment, Reactive Response, and Sensory Orientation, there were wide inter-individual variations, but with mean values in the upper range (Table 3) (Additional file 1). For the control system Stability in Gait, all participants scored in the upper half of the scale. More than 75% of the participants were assessed as having normal performance in 4 of the 5 items in Stability in Gait, whereas only 10 (16%) of the 62 participants were assessed as having normal performance in the last dual task (timed-up-and-go test together with counting backward by 3 starting from 98). This dual task was difficult for most participants regardless of balance group.

**Table 3:** Responses at the Mini-BESTest, the 4 control systems and each of the 14 items in the test. (Group 2 = often balance problems, Group1 = sometimes balance problems, Group 0 = no balance problems)

Variables	All patients (*N* = 62)		Group 2 (*n* = 19)		Group 1 (*n* = 29)		Group 0 (*n* = 14)	
	Mean (SD)	Min_Max	Mean (SD)	Min_Max	Mean (SD)	Min_Max	Mean (SD)	Min_Max
Mini-BESTest, 0–28	22.8 (3.5)	13–38	20.5 (4.2)	13–28	23.5 (2.8)	16–28	24.4 (2.1)	21–28
I Anticipatory Adjustment, 0–6	4.5 (1.2)	2–6	3.8 (1.4)	2–6	4.6 (1.0)	3–6	5.1 (0.5)	4–6
II Reactive Response, 0–6	4.3 (1.7)	0–6	3.8 (1.6)	0–6	4.6 (1.8)	0–6	4.6 (1.5)	2–6
III Sensory Orientation, 0–6	5.5 (0.8)	2–6	5.0 (1.1)	2–6	5.7 (0.6)	4–6	5.9 (0.3)	5–6
IV Stability in Gait, 0–10	8.5 (1.1)	6–10	8.0 (1.4)	6–10	8.6 (0.9)	7–10	8.7 (0.8)	7–10

There was large inter-individual variation within each of the 3 balance groups for most of the tests in the 4 control systems. There was a tendency, however, towards smaller proportions of participants in Group 2 with normal performance compared to the other 2 groups (Table 3). For the sub-scores for 2 control systems (Anticipatory Adjustment and Sensory Orientation), significant statistical differences were found between the 3 groups (*P* ≤ .002). Female participants responded more poorly than males (− 1.0 (95% CI 1.9–0.2)), *P* = .016 in the Reactive Response control system. Only a small fraction of the participants showed poor performance on the individual tests in the Mini-BESTest.

### Associations between self-reported balance problems and test results

The Mini-BESTest showed a statistically significant association with self-reported balance problems as assessed by Balance_2 (sometimes or no balance problems versus frequent balance problems) (Rho = 0.39, *P* = .002) (Table 4). Significant associations were also seen between Balance_2 and 2 of the control systems: Anticipatory Adjustment (Rho = 0.33, *P* = .008) and Sensory Orientation (Rho = 0.45, *P* < .001).

**Table 4:** Bivariate correlations (Spearman’s Rho) between the Mini-BESTest, disability measures and age, gender and BMI

Variables	Balance_2	FUNC	ODI	Age	Gender	BMI
FUNC	−.13	1		.02	−.18	.29^a^
ODI	−.20	.74^b^	1	.03	−.24	.28^a^
Mini-BESTest	.39^b^	.33^b^	.37^b^	.43^b^	.27^a^	−.12
I. Anticipatory adjustment	.33^b^	−.19	−.17	−.34^a^	.05	−.25^a^
II. Reactive response	.23	−.24	−.15	.38^†^	.30^a^	−.02
III. Sensori-orientation	.45^b^	−.23	−.23	−.28^a^	.02	−.16
IV. Walking stability	.23	−.24	.43^b^	−.23	−.26^a^	.06

^a^Correlation is significant at the 0.05 level (2-tailed)

^b^Correlation is significant at the 0.01 level (2-tailed) *Balance_2* Sometimes and no balance problems (coded 1) versus often balance problems (coded 0) *FUNC* Spinal Stenosis Questionnaire, physical functioning, *ODI* Oswestry Disability Index, *BMI* Body Mass Index (kg/m2)

The logistic regression models, adjusted for age, gender, and BMI, supported the associations shown above. The strongest association was seen between Sensory Orientation and Balance_2, implying that it is 4.4 times more likely that persons would have no or occasional balance problems with each unit of increase in Sensory Orientation (Table 5).

**Table 5:** Associations between Balance_2 (Sometimes and no balance problems versus often balance problems)) as the dependent variable and the 4 subsystems in the Mini-BESTest. Crude and adjusted logistic regression analyses

	Balance_2			
	Crude		Adjusted^a^	
Variables	OR (95%CI)	*p*-value	OR (95%CI)	*p*-value
Mini-BESTest	1.3 (1.1,1.6)	.002	1.6 (1.2, 2.0)	.001
I. Anticipatory adjustment	2.2 (1.3,3.7)	.005	2.3 (1.2,4.1)	.008
II. Reactive response	1.3 (0.9,1.8)	.128	1.4 (1.0,2.1)	.074
III. Sensori-orientation	3.7 (1.6, 8.4)	.002	4.4 (1.8,11.0)	.001
IV. Walking stability	1.8 (1.1, 3.1)	.031	2.3 (1.2,4.4)	.010^b^

^a^adjusted by age, gender and BMI. ^b^BMI significant covariate OR(95%CI) = 0.9(0.8,1.0) *P* = .044

Balance_2 = Sometimes and no balance problems (coded 1) versus often balance problems (coded 0)

### Associations between self-reported function and disability, and test results

FUNC and ODI were significantly associated with the total Mini-BESTest (Table 4). An association was also found between Stability in Gait and ODI (Rho = − 0.43, *P* < .001), whereas the correlations between FUNC and the 4 control systems were all poor and not statistically significant (Rho’s ≤ − 0.24, *P* > .056).

When adjusted linear regression analyses were performed, using respectively FUNC and ODI as dependent variables, the Mini-BESTest was only significantly associated with FUNC (B = − 0.1(95%CI -0.1, − 0.02), *P* = .042). The participants dynamic balance explained 16.7% of the variation in FUNC (r^2^ = 0.167) adjusted for age, gender and BMI. The control system Stability in Gait showed significant association with ODI in the adjusted models, with BMI as a significant covariate (Table 6).

**Table 6:** Associations between disability measures, respectively FUNC and ODI, as dependent variables and the control systems in the Mini-BESTest. Crude and adjusted linear regression analyses

	FUNC				ODI			
Variables	Crude		Adjusted^a^		Crude		Adjusted^a^	
	B (95%CI B)	*p*-value	B (95%CI)	*p*-value	B (95%CI)	*p*-value	B (95%CI)	*p*-value
Mini-BESTest	−0.1(−0.1,-0.01)	.015	− 0.1(− 0.1,-0.02)	.042	−1.1(−2.1,-0.1)	.030	− 0.9 (− 2.0,0.2)	.098
I Anticipatory Adjustment	− 0.1(0.3,0.01)	.055	− 0.1(0.2,0.1)	.190	− 1.6 (−4.6,1.4)	.282	− 0.6(−3.9,2.6)	.704
II Reactive Response	− 0.1 0.2,0.01)	.057	− 0.1(− 0.2, − 0.1)	.126^b^	−1.0 (− 3.3,1.0)	.312	−0.5 (− 2.7,1.8)	.680
III Sensory Orientation	−0.1 (0.3,0.1)	.348	−0.1(− 0.3,0.1)	.552^†^	−2.6 (−6.8,1.6)	.216	− 1.8 (− 6.1,2.4)	.390
IV Stability in Gait	− 0.1 (0.3,0.01)	.053	− 0.1(− 0.3,0.1)	.065^†^	−5.1 (−8.1,-2.2)	.001	−5.3 (−8.3,-2.2)	.001^b^

^a^adjusted by with age, gender and BMI. ^b^BMI significant (0.020 ≤ *P* ≤ 0.041) *FUNC* Spinal Stenosis Questionnaire; physical functioning, *ODI* Oswestry Disability Index

## Discussion

The results of the present study show that participants with LSS have large inter-individual variation in balance as assessed by the Mini-BESTest, although most of the participants displayed rather good dynamic balance. The participants who reported having frequent balance problems had poorer dynamic balance than the participants who reported sometimes or no balance problems. Self-reported balance problems were significantly associated with poorer scores on the Mini-BESTest, as was the case for the control systems; Anticipatory Adjustment, Sensory Orientation, and Walking Stability in Gait. The Mini-BESTest was weak, though statistical significantly associated with self-reported physical function (FUNC).

The participants with LSS in the present study had almost identical mean scores (22.8) on the Mini-BESTest compared to healthy Canadian adults (mean 22.9 for the age group 60–80) [26] . The participants without balance problems in our study had better balance capacity (mean 24.4) than the Canadian reference group, whereas those who experienced balance problems had lower scores (mean 20.5). Among persons with Parkinson’s disease of comparable age to the participants in the present study, fallers and non-fallers scored 14 (SD 6.2) and 23 (SD 5.5) points, respectively, on the Mini-BESTest. [41] These results show that persons with lumbar spinal stenosis do not necessarily have impaired balance when tested with the Mini-BESTest.

Even though the Mini-BESTest scores for the participants with balance problems were lower than for the other 2 groups, these participants also showed large inter-individual variation in balance capacity. As shown in Fig. 2, maximum scores were found in all groups, and at least 50% of participants in all 3 groups scored above the threshold for impaired postural response [29]. Similarly, Leddy and co-workers found large variation among both the fallers and non-fallers among persons with Parkinson’s disease [41]. Our results also showed large variability for the subsystems, underlining that persons with LSS constitute a heterogeneous group with regard to dynamic balance. We used the balance question from the disease specific SSQ, asking for frequency of balance problems. The test results showed large heterogeneity in the group of participants with frequent balance problems and may imply that this question was unprecise. One additional question of severity or fall-tendency could probably have nuanced the description of the balance problems and shown stronger association with the test results.

The results in the present study suggest that the Mini BESTest could provide useful information about balance in persons with LSS, important for clinicians, and thus improve the clinicians ability to identify specific control systems that contribute to limitation of individual balance capacity. This information might be helpful in improving or developing more targeted physiotherapy interventions. As the test battery does not require special equipment and can be completed in an acceptable time, makes it usable in clinical settings and individualized and person-centred rehabilitation interventions can be achieved.

Walking-induced pain and balance problems have been presented as the primary factors limiting ambulation in persons with LSS. [42] The associations between balance responses and self-reported measures investigated in this study have elucidated this further. The disease-specific FUNC, which mainly reflects degree of pain while walking, was associated with the total Mini-BESTest and not specifically with any of the control systems, which suggests that walking limitations in persons with LSS may be related to several balance control systems. The subsystem Stability in Gait was significantly associated with both balance problems and ODI. This corresponds well with the study by Passmore and co-workers, which showed that, for persons with symptomatic LSS, complementary movements are challenging [14].

Lumbar spinal stenosis has both anatomical and clinical definitions. We decided to use a disease-specific questionnaire to reveal the participants clinical status rather than the degree spine degeneration. The relationship between clinical symptoms or disability and the degree of radiologically verified constriction in the spinal canal is still unclear [43, 44].

### Study limitations

The present study has some limitations. Measurement of balance by performance tests such the Mini-BESTest will always include an intra-individual variation. Hence, the tests may not always capture the typical condition for the patient. There will also be a certain variation in judgement of performance by the assessors. The latter was reduced as much as possible by training of the raters. To ensure the quality of the evaluation of the balance performances and diminishing the variation in the observations, the three assessors were trained in their observation skills and in using the test protocol. Another limitation is the small sample size. A larger number of participants would have provided more robust results and enabled us to perform other sub-group analyses, as well as more in-depth analyses of the balance responses. With a larger sample, multiple factors as MRI- findings, EQ5D and HSCL25 could have been entered adjusted regression analyses and explained more of the variation in the dependent variables.

## Conclusions

The Mini-BESTest revealed a large variation in dynamic balance from very good to poor in persons with LSS. The test results were associated with the participants’ self-reported balance problems and with walking function (FUNC), although a large fraction of the participants displayed to have good balance. The performance test thus appears to provide additional information to self-reported balance problems in persons with LSS. By identifying different kinds of balance limitations, the Mini-BESTest could provide results useful for physiotherapists working with person-centered rehabilitation in persons with LSS.

## Additional file

Additional file 1: **Table S1**. Responses at each of the 14 items in the Mini-BESTest (*N* = 62). (Group 2 = often balance problems, Group1 = sometimes balance problems, Group 0 = no balance problems). (PDF 285 kb)

## Abbreviations

BMI: Body Mass Index (kg/m^2^)
FUNC: Spinal stenosis questionnaire, physical function
NRS: Numeric rating scale
ODI: Oswestry Disability Index
SYMP: Spinal stenosis questionnaire, symptom severity

## References

[1] Ciol MA, Deyo RA, Howell E, Kreif S (1996). An assessment of surgery for spinal stenosis: time trends, geographic variations, complications, and reoperations. J Am Geriatr Soc.

[2] Verbiest H (1954). A radicular syndrome from developmental narrowing of the lumbar vertebral canal. J Bone Joint Surg Br.

[3] Atlas SJ, Delitto A. Spinal stenosis: surgical versus nonsurgical treatment. Clin Orthop Relat Res. 2018;40(2):232-237. 10.1097/01.blo.0000198722.70138.96 16462443

[4] Stucki G, Liang MH, Fossel AH, Katz JN (1995). Relative responsiveness of condition-specific and generic health status measures in degenerative lumbar spinal stenosis. J Clin Epidemiol.

[5] Lin SI, Lin RM (2005). Disability and walking capacity in patients with lumbar spinal stenosis: association with sensorimotor function, balance, and functional performance. J Orthop Sports Phys Ther.

[6] Thornes E, Robinson HS, Vollestad NK. Degenerative lumbar spinal stenosis and physical functioning: an exploration of associations between self-reported measures and physical performance tests. Disabil Rehabil. 2016:1–6. 10.1080/09638288.2016.1250123 27846739

[7] Lurie J, Tomkins-Lane C (2016). Management of lumbar spinal stenosis. Bmj.

[8] Tomkins-Lane CC, Battie MC (2013). Predictors of objectively measured walking capacity in people with degenerative lumbar spinal stenosis. J Back Musculoskelet Rehabil.

[9] Iversen MD, Kale MK, Sullivan JT (2009). Pilot case control study of postural sway and balance performance in aging adults with degenerative lumbar spinal stenosis. J Geriatr Phys Ther.

[10] Katz JN, Dalgas M, Stucki G, Katz NP, Bayley J, Fossel AH, Chang LC, Lipson SJ (1995). Degenerative lumbar spinal stenosis. Diagnostic value of the history and physical examination. Arthritis Rheum.

[11] Truszczynska A, Drzal-Grabiec J, Trzaskoma Z, Rapala K, Tarnowski A, Gorniak K (2014). A comparative analysis of static balance between patients with lumbar spinal canal stenosis and asymptomatic participants. J Manip Physiol Ther.

[12] Krebs DE, Goldvasser D, Lockert JD, Portney LG, Gill-Body KM (2002). Is base of support greater in unsteady gait?. Phys Ther.

[13] Kim HJ, Chun HJ, Han CD, Moon SH, Kang KT, Kim HS, Park JO, Moon ES, Kim BR, Sohn JS (2011). The risk assessment of a fall in patients with lumbar spinal stenosis. Spine (Phila Pa 1976).

[14] Passmore SR, Johnson M, Pelleck V, Ramos E, Amad Y, Glazebrook CM (2014). Lumbar spinal stenosis and lower extremity motor control: the impact of walking-induced strain on a performance-based outcome measure. J Manip Physiol Ther.

[15] Backstrom KM, Whitman JM, Flynn TW (2011). Lumbar spinal stenosis-diagnosis and management of the aging spine. Man Ther.

[16] Englund J (2007). Lumbar spinal stenosis. Curr Sports Med Rep.

[17] Kreiner DS, Shaffer WO, Baisden JL, Gilbert TJ, Summers JT, Toton JF, Hwang SW, Mendel RC, Reitman CA, North American Spine S (2013). An evidence-based clinical guideline for the diagnosis and treatment of degenerative lumbar spinal stenosis (update). Spine J.

[18] Djurasovic M, Glassman SD, Carreon LY, Dimar JR (2010). Contemporary management of symptomatic lumbar spinal stenosis. Orthop Clin North Am.

[19] Jones KD, King LA, Mist SD, Bennett RM, Horak FB (2011). Postural control deficits in people with fibromyalgia: a pilot study. Arthritis Res Ther.

[20] Anson E, Thompson E, Odle BL, Jeka J, Walls ZF, Panus PC. Influences of age, obesity, and adverse drug effects on balance and mobility testing scores in ambulatory older adults. J Geriatr Phys Ther. 2017; 10.1519/JPT.0000000000000124 PMC5503816 28079635

[21] Hutzler Y, Korsensky O, Laufer Y. Rapid stepping test towards virtual visual objects: feasibility and convergent validity in older adults. Technol Health Care. 2017. Epub ahead of print. 10.3233/THC-161251 27589506

[22] Frih B, Mkacher W, Jaafar H, Frih A, Ben Salah Z, El May M, Hammami M. Specific balance training included in an endurance-resistance exercise program improves postural balance in elderly patients undergoing haemodialysis. Disabil Rehabil. 2018;40(7):784-237. 10.1080/09638288.2016.1276971 28084833

[23] Bacha JMR, Gomes GCV, de Freitas TB, Viveiro LAP, da Silva KG, Bueno GC, Varise EM, Torriani-Pasin C, Alonso AC, Luna NMS (2018). Effects of Kinect adventures games versus conventional physical therapy on postural control in elderly people: a randomized controlled trial. Games Health J.

[24] Franchignoni F, Horak F, Godi M, Nardone A, Giordano A (2010). Using psychometric techniques to improve the balance evaluation systems test: the mini-BESTest. J Rehabil Med.

[25] Thornes E, Ikonomou N, Grotle M (2011). Prognosis of surgical treatment for degenerative lumbar spinal stenosis: a prospective cohort study of clinical outcomes and health-related quality of life across gender and age groups. Open Orthop J.

[26] O'Hoski S, Winship B, Herridge L, Agha T, Brooks D, Beauchamp MK, Sibley KM (2014). Increasing the clinical utility of the BESTest, mini-BESTest, and brief-BESTest: normative values in Canadian adults who are healthy and aged 50 years or older. Phys Ther.

[27] Horak FB, Wrisley DM, Frank J (2009). The balance evaluation systems test (BESTest) to differentiate balance deficits. PhysTher.

[28] King L, Horak F (2013). On the mini-BESTest: scoring and the reporting of total scores. Phys Ther.

[29] King LA, Priest KC, Salarian A, Pierce D, Horak FB (2012). Comparing the mini-BESTest with the berg balance scale to evaluate balance disorders in Parkinson's disease. Parkinsons Dis.

[30] Godi M, Franchignoni F, Caligari M, Giordano A, Turcato AM, Nardone A (2013). Comparison of reliability, validity, and responsiveness of the mini-BESTest and berg balance scale in patients with balance disorders. Phys Ther.

[31] Di Carlo S, Bravini E, Vercelli S, Massazza G, Ferriero G (2016). The mini-BESTest: a review of psychometric properties. Int J Rehabil Res.

[32] Stucki G, Daltroy L, Liang MH, Lipson SJ, Fossel AH, Katz JN (1996). Measurement properties of a self-administered outcome measure in lumbar spinal stenosis. Spine (Phila Pa 1976).

[33] Fairbank JC, Couper J, Davies JB, O'Brien JP (1980). The Oswestry low back pain disability questionnaire. Physiotherapy.

[34] Stokes OM, Cole AA, Breakwell LM, Lloyd AJ, Leonard CM, Grevitt M (2017). Do we have the right PROMs for measuring outcomes in lumbar spinal surgery?. Eur Spine J.

[35] EuroQol Group. EuroQol--a new facility for the measurement of health-related quality of life. Health Policy. 1990;16(3):199-208. 10.1016/0168-8510(90)90421-9 10109801

[36] DeRogatis LR, Lipman RS, Rickels K, Uhlenhuth EH, Covi L (1974). The Hopkins symptom checklist (HSCL). A measure of primary symptom dimensions. Mod Probl Pharmacopsychiatry.

[37] Pallant J (2007). SPSS Survival Manual, Third edn.

[38] Winter D (1995). Human balance and posture control during standing and walking. Gait Posture.

[39] Sturnieks DL, St GR, Lord SR (2008). Balance disorders in the elderly. Neurophysiol Clin.

[40] Knutsson B, Sanden B, Sjoden G, Jarvholm B, Michaelsson K (2015). Body mass index and risk for clinical lumbar spinal stenosis: a cohort study. Spine(Phila Pa 1976).

[41] Leddy AL, Crowner BE, Earhart GM (2011). Utility of the mini-BESTest, BESTest, and BESTest sections for balance assessments in individuals with Parkinson disease. J Neurol Phys Ther.

[42] Iversen MD, Katz JN (2001). Examination findings and self-reported walking capacity in patients with lumbar spinal stenosis. Phys Ther.

[43] Kalichman L, Cole R, Kim DH, Li L, Suri P, Guermazi A, Hunter DJ (2009). Spinal stenosis prevalence and association with symptoms: the Framingham study. Spine J.

[44] Sirvanci M, Bhatia M, Ganiyusufoglu KA, Duran C, Tezer M, Ozturk C, Aydogan M, Hamzaoglu A (2008). Degenerative lumbar spinal stenosis: correlation with Oswestry disability index and MR imaging. Eur Spine J.

