# Supplementary material for: Dynamic balance in patients with degenerative lumbar spinal stenosis; a cross-sectional study
Source: BMC Musculoskelet Disord. 2018 Jun 15;19:192. doi: 10.1186/s12891-018-2111-x (PMC6003037; doi:10.1186/s12891-018-2111-x)
Supplement: Supplementary file 1 — Table S1. Responses at each of the 14 items in the Mini-BESTest (N = 62). (Group 2 = often balance problems, Group1 = sometimes balance problems, Group 0 = no balance problems). (PDF 285 kb) [file 12891_2018_2111_MOESM1_ESM.pdf]

Variables	All patients (N=62)			Group 2 (n=19)			Group 1 (n=29)			Group 0 (n=14)		
	*Poor	Moderate	Normal	Poor	Moderate	Normal	Poor	Moderate	Normal	Poor	Moderate	Normal
**I. Anticipatory Adjustment**												
1.Sit-to-stand	0	1	61	0	0	19	0	1	18	0	0	14
2.Rise-to-toes	9	23	30	6	6	7	3	12	14	0	5	9
3.Stand on one leg, timed to 20s	9	36	17	7	10	2	1	20	8	1	6	7
**II. Reactive Response**												
4.Compensatory stepping correction forward	3	20	39	2	6	11	1	9	19	0	5	9
5.Compensatory stepping correction backward	5	29	28	2	10	7	3	11	15	0	8	6
6.Compensatory stepping correction lateral	6	26	30	2	13	4	3	8	18	1	5	8
**III. Sensory Orientation**												
7.Stance on firm surface, eyes open	0	1	61	0	1	18	0	0	29	0	0	14
8.Stance on foam, eyes closed	4	14	44	3	8	8	1	5	23	0	1	13
9.Stance on incline, eyes closed	1	4	57	1	3	15	0	1	28	0	0	14
**IV. Stability in Gait**												
10.Change in gait speed	0	3	59	0	1	18	0	1	28	0	1	13

11.Walk with head turns, horizontal	2	14	46	1	8	10	1	4	24	0	2	12
12. Walk with pivot turns	0	13	49	0	9	10	0	3	26	0	1	13
13. Step over obstacles	0	4	58	0	1	18	0	1	28	0	2	12
14. Time Up Go, dual task	6	46	10	3	11	5	3	23	3	0	12	2
